# Sulfate alters aerosol absorption properties in East Asian outflow

**DOI:** 10.1038/s41598-018-23021-1

**Published:** 2018-03-26

**Authors:** Saehee Lim, Meehye Lee, Sang-Woo Kim, Paolo Laj

**Affiliations:** 1 Dept. of Earth and Environmental Sciences, Korea University, 02841 Seoul, South Korea; 2 School of Earth and Environmental Sciences, Seoul National University, 08826 Seoul, South Korea; 3 Univ. Grenoble-Alpes, IGE, CNRS, IRD, Grenoble INP, 38000 Grenoble, France; 4 Department of Physics, University of Helsinki, 00014 Helsinki, Finland; 5 Institute of Atmospheric Science and Climate (ISAC)-CNR, 40129 Bologna, Italy

## Abstract

Black carbon (BC) and brown carbon (BrC) aerosols that are released from the combustion of fossil fuels and biomass are of great concern because of their light-absorbing ability and great abundance associated with various anthropogenic sources, particularly in East Asia. However, the optical properties of ambient aerosols are dependent on the mixing state and the chemical composition of absorbing and non-absorbing aerosols. Here we examined how, in East Asian outflows, the parameters of the aerosol optical properties can be altered seasonally in conjunction with the mixing state and the chemical composition of aerosols, using 3-year aerosol measurements. Our findings highlight the important role played by sulfate in East Asia during the warm season in both enhancing single scattering albedo (SSA) and altering the absorption properties of aerosols—enhancing mass absorption cross section of BC (MAC_BC_) and reducing MAC of BrC (MAC_BrC,370_). Therefore we suggest that in global radiative forcing models, particular attention should be paid to the consideration of the accurate treatment of the SO_2_ emission changes in the coming years in this region that will result from China’s air quality policy.

## Introduction

The climate effect of carbonaceous aerosols is of great concern because of their great abundance associated with various anthropogenic sources^1,2^. Emissions of black carbon (BC), the primary absorbing aerosol, increased by almost 50% between 2000 and 2010 in China^3^. Furthermore, East Asia is the region with the greatest emissions of SO_2_^2,3^ and anthropogenic organic carbon (OC), including brown carbon (BrC), which together with BC account for almost 60% of aerosol optical depth (AOD)^4^. As of 2010, Chinese emissions of BC and sulfate were estimated to explain 14% and 28% of current global radiative forcing (RF), respectively^5^, showing the critical impact of BC and sulfate from East Asia on the climate.

Absorbing carbonaceous aerosols and non-absorbing inorganic aerosols such as sulfate are often internally mixed upon atmospheric aging processes while being transported^6,7^. The optical properties of bulk aerosols are, therefore, likely to be altered, leading to uncertainties in the estimation of aerosol forcing. It is thus crucial to understand the intimate link between optical properties and the chemical composition of aerosols in the ambient air to assess their climate forcing more accurately. In East Asia, however, long-term observations of the chemical composition of aerosols in conjunction with their optical properties are particularly scarce. We thoroughly examined how their optical properties are affected by their chemical composition in the outflow region of emissions from China. We used a time series of the light absorption and scattering properties and chemical composition of aerosols sampled at Gosan Climate Observatory (GCO, 33.17°N, 126.10°E, 70 m ASL; shown in Supplementary Fig. S1) during 2008–2010.

The study region is under the influence of the Asian monsoon, leading to dynamic changes in meteorological conditions from season to season (Supplementary Table S1 and Fig. S1). Westerlies are prevalent in the cold season, which efficiently carry continental outflows out to the Western Pacific region. Summer usually starts with heavy rain, and wind shifts from northerly to southerly, bringing air from the Pacific Ocean. During the period of transition to summer, air masses tend to be stagnant, which often causes pollution episodes such as hazes. Considering the noticeable seasonal changes^8^, measurement results are discussed in seasonal groups: cold (Oct.–Mar.) and warm (Apr.–Sep.; April and May [spring] and June to September [summer]).

## Results

**Figure 1:**
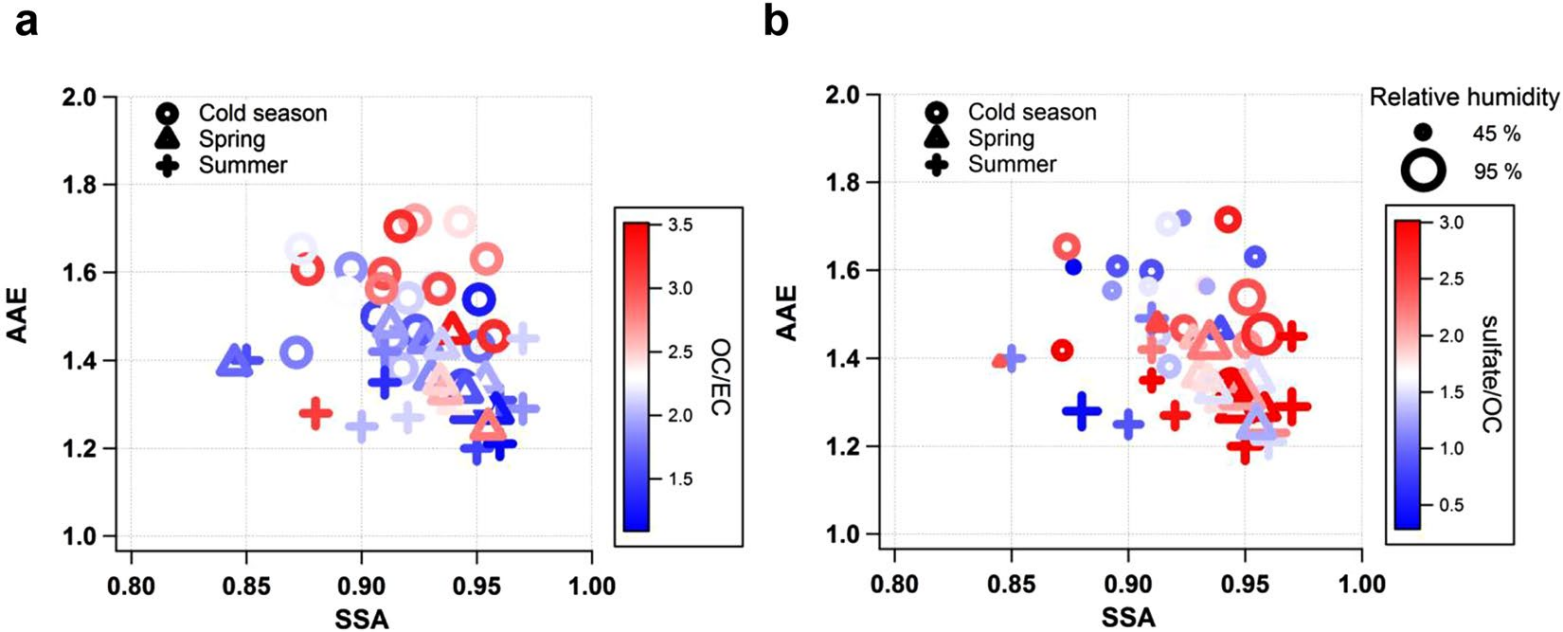
Relationship between single scattering albedo (SSA) and absorption Ångström exponent (AAE) of aerosol measured at GCO from 2008 to 2010. The data are classified by season and color coded by (**a**) OC-to-EC ratio; (**b**) sulfate-to-OC ratio with relative humidity (RH) in different size. The SSA and AAE are derived from measurements at 520 nm and 7 wavelengths between 370 nm–950 nm, respectively (Supplementary Text S3).

On the basis of the optical measurements at seven wavelengths from 370 nm to 950 nm (Supplementary Text S3), we calculated the absorption Ångström exponent (AAE) at 370 nm–950 nm and the single scattering albedo (SSA) at 520 nm (Table 1). These optical properties as well as major chemical constituents show seasonal difference (P < 0.0001 for AAE; Supplementary Fig. S2). Roughly, the SSAs and AAEs were inversely related at GCO (Fig. 1), in which the two regimes are in contrast: one with low SSA (<0.9) and high AAE (>1.5) and the other with high SSA (>0.9) and low AAE (<1.5). While the SSA of GCO was high throughout the year with a mean of 0.93 ± 0.03, it was greater than 0.95 when marine air masses were dominant in summer or continental air was stagnant over the Yellow Sea in late spring^8,9^. Our results are comparable with those of previous studies on East Asia (Supplementary Fig. S3a). Clearly, our mean SSA was much higher than that of Beijing in the visible ranges (0.84)^10,11^ and the annual mean SSA of China (0.89)^12^. By contrast, our mean AAE was similar to that of Beijing in the warmer months^10,11^, when aerosols were less absorbing than the other seasons^13^. This comparison clearly indicates more scattering but slightly less absorbing properties of aerosols at GCO than those at Beijing.

**Table 1:** Summary (mean and 1σ in parentheses) of aerosol measurements at Gosan Climate Observatory (GCO) for the entire period (2008–2010) and for three seasonal categories.

	All	Cold Season	Warm Season	
		October–March	Spring April–May	Summer June–September
**Optical properties**				
SSA^a^	0.93 (0.03)	0.92 (0.03)	0.93 (0.03)	0.93 (0.04)
AAE^1**,b^	1.4 (0.2)	1.6 (0.1)	1.4 (0.1)	1.3 (0.2)
AAE_non-BC_^2**,b^	3.1 (0.3)	3.4 (0.2)	3.0 (0.3)	3.0 (0.4)
MAC_BC_ (m^2^ g^−1^)^2*,a^	5.6 (2.6)	5.0 (1.4)	6.4 (2.9)	5.7 (2.1)
MAC_BrC,370_ (m^2^ g^−1^)	1.2 (0.6)	1.2 (0.3)	1.2 (1.6)	1.2 (0.5)
*σ*_*ap, 520*_ (Mm^−1^)	9.7 (7.6)	9.3 (6.3)	12.1 (10.3)	6.4 (2.5)
*σ*_*sp, 520*_ (Mm^−1^)	135.1 (92.8)	132.2 (101.4)	153.8 (73.4)	119.8 (100.1)
**Chemical properties**				
OC/EC^2*,c^	2.3 (0.7)	2.5 (0.7)	2.1 (0.6)	2.0 (0.8)
Sulfate/OC^2*,c^	2.0 (1.3)	1.4 (0.8)	2.5 (1.4)	2.3 (1.5)
Sulfate/EC^1*,c^	3.9 (1.9)	3.3 (1.7)	4.9 (2.0)	4.1 (2.1)
[NH_4_]/2[SO_4_]^c,d^	0.9 (0.5)	1.0 (0.6)	0.7 (0.1)	0.9 (0.1)
PM_1_ (µg m^−3^)	15.8 (11.6)	13.4 (8.4)	21.8 (15.7)	12.4 (7.3)
PM_10_ (µg m^−3^)	33.5 (24.8)	32.5 (29.4)	41.0 (21.3)	25.0 (10.3)
Sulfate (µg m^−3^)^c^	4.84 (3.53)	4.07 (2.91)	6.60 (4.36)	3.92 (2.58)
OC (µg m^−3^)^c^	2.78 (1.59)	3.08 (1.58)	2.94 (1.86)	1.77 (0.55)
EC (µg m^−3^)^c^	1.30 (0.80)	1.36 (0.86)	1.43 (0.89)	0.93 (0.31)

*^,^**Stars denote the level of significance obtained from the one-way analysis of variance (ANOVA) for estimated optical properties and chemical ratios (*p < 0.05 and **p < 0.0001).

^1^Statistically significant among three seasonal categories.

^2^Statistically insignificant between spring and summer.

^a^Both SSA and MAC_BC_ are calculated at 520 nm, except for SSA estimated at 370 nm in Fig. 3b.

^b^AAE and AAE_non-BC_ are calculated for wavelengths between 370 and 950 nm. “AAE” and “AAE _non-BC_” indicate the AAE of bulk particulate matter and non-BC particulate matter, respectively.

^c^For inorganic water-soluble ions, PM_1_ data is used.

^d^Data available only for 2008 because of limited ammonium (NH_4_^+^) data.

**Figure 2:**
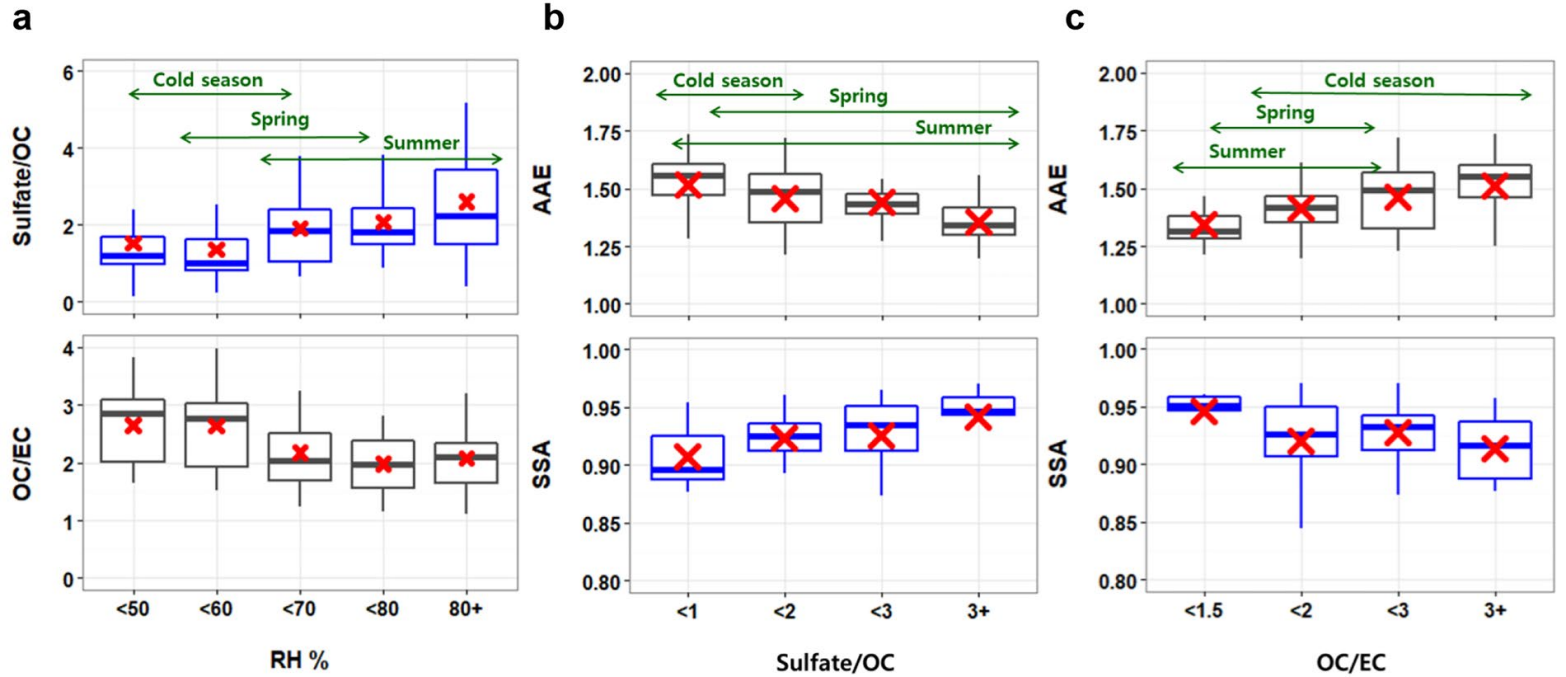
Relationship between chemical composition and optical properties of aerosol. (**a**) The variation in sulfate-to-OC ratio and OC-to-EC ratio as a function of ambient RH; The variation in SSA and AAE as a function of (**b**) sulfate-to-OC ratio; (**c**) OC-to-EC ratio. Seasonal ranges indicated by arrows correspond to mean ± 1σ. SSA and AAE are the same as those shown in Fig. 1.

The relative abundance of major chemicals in the composition, including sulfate, OC, and elemental carbon (EC), was well classified in the SSA–AAE domain (Fig. 1a and b). While OC-to-EC ratios were relatively high in the region of low SSA and high AAE (Fig. 1a), sulfate-to-OC ratios showed the opposite tendency, being higher at high SSA and low AAE (Fig. 1b). In particular, sulfate-to-OC ratios increased, being 70% higher in the warm season than it was in the cold season as the ambient relative humidity (RH) increased from winter to summer; however, OC-to-EC ratios were higher by 20–25% in the cold season (Fig. 2a). In the transition period between the two seasons, stagnant conditions often develop over the Yellow Sea under high pressure with the reduced but substantial influence of continental emissions^8^ (Supplementary Fig. S1 and Table S1). Although SO_2_ emissions reach their maximum in the cold season, their conversion to sulfate would be more efficient via aqueous chemistry under high RH while the air mass is slowly transported over the Yellow Sea in the warm season. The average SSA was 0.95 or even larger, with a sulfate-to-OC ratio of over 3. In East Asia, the fine mode AOD reached the peak in the warm season^8^, and sulfate was attributed to the enhanced fine mode fraction (FMF)^9^. Meanwhile, the light-absorbing OC component, i.e., BrC, possibly explains the increased AAE in the cold season when the continental influence is the greatest (Fig. 2c). These results indicate that in Asian outflows, the optical properties of aerosols were intimately tied to their chemical composition, which largely depended on meteorological conditions that determined aerosol sources and controlled atmospheric processing during transport.

**Figure 3:**
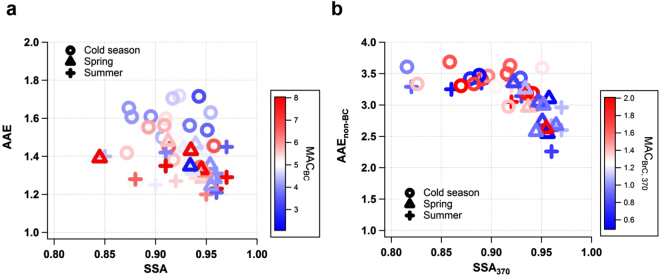
Relationship between SSA and AAE of aerosol shown by mass absorption coefficient (MAC) of BC and BrC according to season. (**a**) The MAC_BC_ values, in which MAC_BC_ is derived at a wavelength of 520 nm (Supplementary Text S4), are color coded in a SSA-AAE domain, which is the same as that shown in Fig. 1; (**b**) The MAC_BrC,370_ values, in which MAC _BrC,370_ is derived at a wavelength of 370 nm (Supplementary Text S4), are color coded in a SSA_370_–AAE_non-BC_ domain, in which SSA is calculated at a wavelength of 370 nm and AAE for non-BC is derived from 7 wavelengths between 370 nm–950 nm.

Absorbing aerosols such as BC and BrC often coexist with other components^7,15^, and the chemical composition of the bulk aerosols and their mixing state directly determine their optical properties. These properties may be reflected in the mass absorption cross-section (MAC) of the absorbing aerosols. We estimated the MAC of BC (MAC_BC_) at 520 nm under the assumption that BC is the only absorbing aerosol in the near-infrared (IR) region^16,17^ and that its absorption is spectrally independent in the visible range^11^. In the present study, mean MAC_BC_ was 5.6 ± 2.6 m^2^ g^−1^ (Table 1), which is similar to those reported at GCO previously^18^ and in rural areas in China^19,20^. Our MAC_BC_ includes absorption by bare BC and lensing enhancement by non-absorbing coating materials^21^ under the assumption that AAE = 1. In addition, the MAC of BrC was estimated at 370 nm (MAC_BrC,370_) by subtracting the absorption of BC and dust from total absorption and normalizing the absorption of BrC by PM_1_ OC mass concentration (Supplementary Text S4). The MAC_BrC,370_ varied from 0.3 to 2.8 at 370 nm (5^th^–95^th^ percentile) with a mean of 1.2 ± 0.6 m^2^ g^−1^ (Table 1). The MAC_BC_ and MAC_BrC,370_ calculated in this study were compared with those of previous studies (Supplementary Fig. S3b). Our MAC_BC_ and MAC_BrC,370_ were lower by approximately a factor of 1.5 than those of the megacities on the eastern coast of China^11,22,23^. The lower MAC at GCO when compared with China may be due to the coagulation of absorbing particles during transport from source areas^24^ and/or less absorption compared to the enhanced absorption at the Chinese megacities by coating with organic aerosols. The estimated MAC_BC_ values at GCO differed seasonally (P < 0.05), and most high values were found in the regime of high SSA and low AAE (Fig. 3a). The seasonal mean MAC_BC_ was higher, with higher sulfate-to-OC ratios in spring (6.4 ± 2.9 m^2^ g^−1^) and summer (5.7 ± 2.1 m^2^ g^−1^), than it was in the cold season (5.0 ± 1.4 m^2^ g^−1^) (Table 1). This also shows that at GCO, the absorption property of aerosols is tightly linked with their chemical composition of bulk aerosols. It has been reported that BC absorption can be enhanced by a coating of secondary aerosols, referred to as the lensing effect^15,21^. In the warm season, the continental outflow is weakened as stagnant conditions develop. Consequently, gaseous precursors and aerosols stay longer over the Yellow Sea, which is a favorable condition for carbonaceous aerosols to be internally mixed with sulfate.

**Figure 4:**
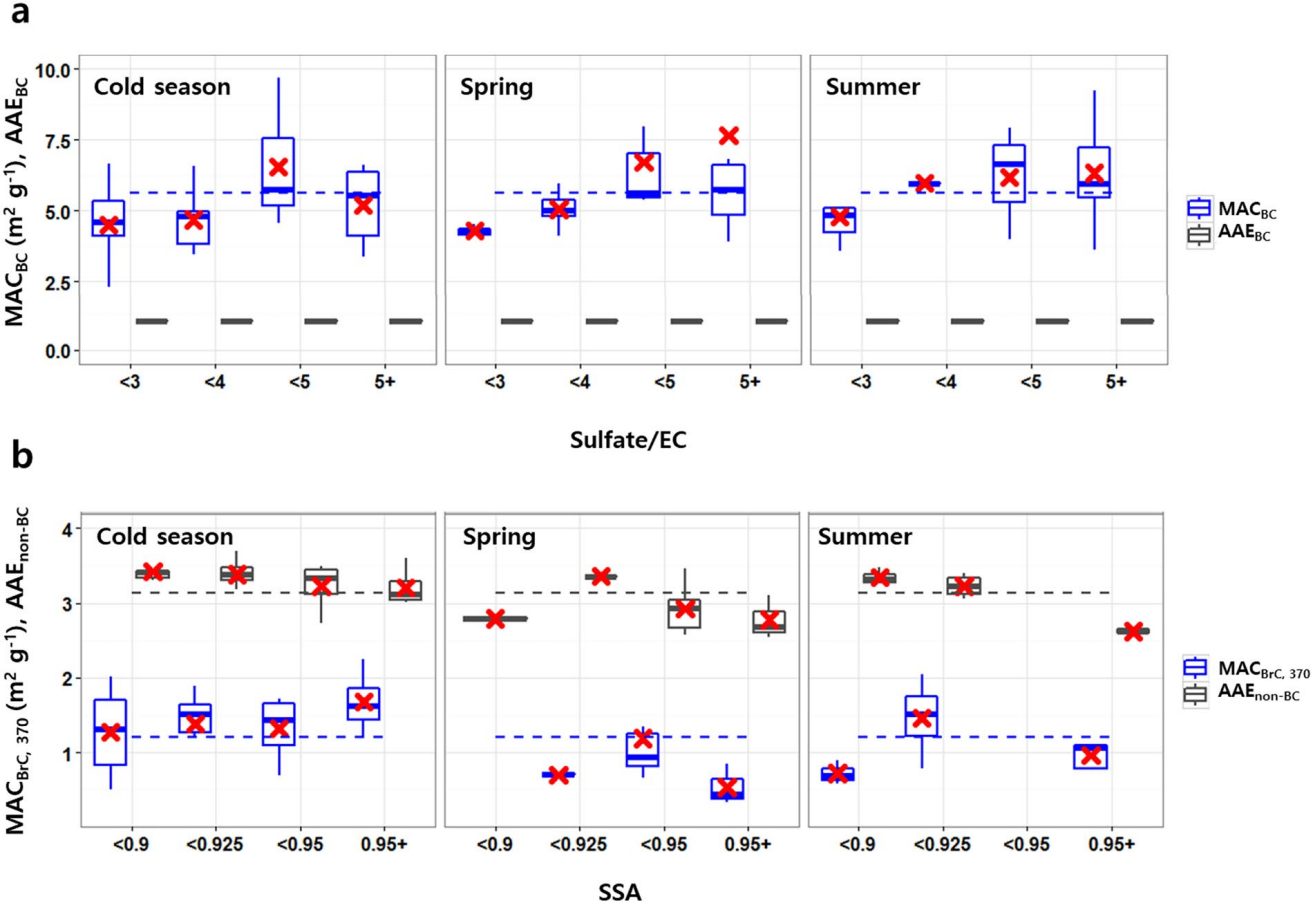
Seasonal characteristics of MAC (MAC_BC_, MAC_BrC,370_) and AAE (AAE_BC_ of 1, AAE_non-BC_) in relation to chemical composition and SSA. (**a**) The relation of MAC_BC_ and AAE_BC_ to sulfate-to-EC ratio; (**b**) The relation of MAC_BrC,370_ and AAE_non-BC_ to SSA. Mean values for the whole period are shown by dotted lines. Red cross indicates a mean value in each bin.

In Fig. 4a, MAC_BC_ tended to increase with the sulfate-to-EC ratio during the warm season, whereas there was a less clear relationship between the two in the cold season. Sulfate-to-EC ratios were higher by 30%–50% in the warm season than they were in the cold season. When the sulfate-to-EC ratio was greater than 5 in spring, the mean MAC_BC_ was enhanced by a factor of 2 relative to the MAC_BC_ with a sulfate-to-EC ratio of below 3. Even though it was not feasible to assess the enhancement factor of BC absorption relative to bare BC (*E*_*abs*_ or *E*_*MAC*_)^7,15^ in this study, the enhancement of our MAC_BC_ is in accordance with measured ranges reported in the literatures (factor of approximately 1.1–2.0). The concurrent increase in MAC_BC_ and sulfate observed in this study is evidence of BC absorption enhancement due mainly to sulfate being internally mixed with BC in the warm season^19^. Moreover, the mean molar ratio of [NH_4_]/2[SO_4_] was at its minimum in spring (Table 1), which is favorable for the hygroscopic growth of aerosols under high RH and acidic conditions^25^.

In contrast to MAC_BC_, MAC_BrC,370_ was lower than the mean (1.2 m^2^ g^−1^) in the regime of high SSA and low AAE (Fig. 3b), with a high sulfate-to-OC ratio (Fig. 1b) during the warm season. Given that biomass-burning OC particles tend to absorb radiation more strongly^13,22^, our high AAE_non-BC_ and MAC_BrC,370_ during the cold season would indicate the influence of biomass combustion in the Asian continent. In the warm season, the decrease in both MAC_BrC,370_ and OC mass fraction suggests the possibility of the chemical transformation of organic components or their partial evaporation during long-range transport across the Yellow Sea. In a pair of companion papers^9,26^, OC mass decreased but OC-to-EC ratios increased in the warm season, indicating secondary formation. Recent studies reported that the organic compounds in biomass burning plumes were rapidly oxidized with physical, chemical, and optical changes during the first few hours of their atmospheric transport^27^. In laboratory and chamber studies, light-absorbing organic compounds were photo-chemically converted to non-absorbing products on the order of hours in a process referred to as photo-bleaching^28,29^. GCO is distant enough from China for BrC to be degraded upon transport, which is consistent with a former study at GCO showing photochemical degradation of organic compounds in summer^30^.

In addition, we hypothesize that sulfate alters the absorption of BrC in internal mixtures by lowering AAE_non-BC_ and MAC_BrC,370_. The MAC_BrC,370_ values were often lower in the warm season (<1.2 in Figs 3b and 4b), less than half the MAC_BrC_ previously estimated at GCO and over the Yellow Sea^18,31^. These MAC_BrC,370_ values were similar to the MAC of water-soluble OC (MAC_WSOC_) of GCO in spring^32^. The sulfate-dominated conditions under high RH were certainly favorable for abundant WSOC^33^ and the internal mixing of aerosols. The optical properties of light-absorbing aerosols are complex functions of core morphology, the type of coating material (absorbing vs. non-absorbing) and its thickness, and the type of internal mixing^24,34^. Therefore, the extent of sulfate coating altering non-BC absorbing particles in internal mixtures and the detailed mechanisms are still poorly understood.

Our 3-year atmospheric measurements at GCO show that in conjunction with chemical properties, the optical properties of aerosols of the East Asian outflow were significantly modified upon transport over the Yellow Sea. These results reveal that in a sulfate-dominated environment, SSA tended to enhance with gradual increases of sulfate as ambient RH increased and the absorption of BC and BrC was altered through the internal mixing of aerosols. The findings of this study highlight the important role played by sulfate in enhancing SSA and MAC_BC_ and reducing MAC_BrC,370_ in East Asia. The complex nature of the state of aerosol mixing in the real atmosphere, including the chemical composition of primary coating materials such as sulfate and BrC, should be considered in regional climate models^1,35^.

Global climate models estimate that Chinese emissions contribute more to the negative global RF (15%) than they do to the positive global RF (12%) while their overall contribution is 10% of the net global RF from anthropogenic emissions (0.30 ± 0.11 W m^−2^ out of 2.88 ± 0.46 W m^−2^)^5^. Our results suggest that there is a stronger negative forcing of sulfate in East Asia, particularly in the warm season when SSA is high (mean = 0.93). In this context, it is noteworthy that Chinese emissions of SO_2_ are expected to be reduced in the coming years through cuts in coal usage in order to improve air quality. Therefore, particular attention should be paid to the accurate estimation of SO_2_ emissions as model uncertainties may be larger because of changes in SO_2_ emissions^5,36^.

## Methods

For the chemical composition of daily filter samples (PM_1_ and PM_10_), water-soluble ions were analyzed by an Ion Chromatography and organic carbon (OC) and elemental carbon (EC) were determined following the Interagency Monitoring of Protected Visual Environments thermal/optical reflectance protocol (i.e., IMP_TOR)^37^. Light scattering and absorption properties of aerosols were measured using an integrating nephelometer (model 3563, TSI Inc., USA) and a seven-wavelength aethalometer at 370 nm–950 nm (AE-31, Magee Scientific Corp., USA), respectively. Further details of aerosol sample collection and analytical methods are provided in Supplementary Text S1 and S2 and our companion papers^9,26^. We performed a field calibration for the aethalometer multi-scattering correction (hereafter denoted by *C*) compared with a Photoacoustic Spectrometer 3-wavelength (PASS-3; 405, 532, and 781 nm) and applied the newly determined wavelength-dependent *C* values (Supplementary Text S3).

To attribute the total light absorption for BC, dust, and BrC absorption, we assumed that the absorption for BC varies with λ^−1^ and that BC is the only significant light absorber at 950 nm because both BrC and dust absorb light weakly in the near-IR^16,17^. We then estimated dust absorption, using a series of MAC of dust (MAC_dust_; 0.087, 0.050, 0.037, 0.027, 0.013, 0.001 m^2^ g^−1^ at 370 nm–880 nm), which was estimated for dust events at a polluted site near Beijing using the absorption predicted by Mie scattering theory and measured dust volume concentration^11^. The dust mass concentration is estimated by supermicron (PM_1–10_) water-soluble calcium, using an Al/dust ratio in a Chinese loess-certified reference material (CRM) and a water-soluble Ca^2+^/Al (=0.24) ratio reported in a previous experiment conducted at GCO^38^. The absorption of BrC was then derived by subtracting the absorption of BC and dust from the total absorption at all wavelengths, and the absorption of BrC was normalized by PM_1_ OC mass concentration, resulting in MAC of BrC (MAC_BrC_) at all wavelengths for each daily sample. The AAE of non-BC absorbing particles (AAE_non-BC_) was calculated at 370 nm–950 nm by subtracting the absorption of BC from total absorption (Supplementary Text S4). See ‘Availability of materials and data’ section for more information.

### Availability of materials and data

The datasets generated during and/or analysed during the current study are available from the corresponding author on reasonable request.

## Electronic supplementary material

Supplementary information
