# Supplementary material for: Sulfate alters aerosol absorption properties in East Asian outflow
Source: Sci Rep. 2018 Mar 26;8:5172. doi: 10.1038/s41598-018-23021-1 (PMC5980100; doi:10.1038/s41598-018-23021-1)
Supplement: Supplementary file 1 — Supplementary information [file 41598_2018_23021_MOESM1_ESM.pdf]

Sulfate alters aerosol absorption properties in East Asian outflow

Saehee Lim[1], Meehye Lee[1, *], Sang-Woo Kim[2] and Paolo Laj[3, 4, 5]

[1]Dept. of Earth and Environmental Sciences, Korea University, 02841 Seoul, South Korea.

[2]School of Earth and Environmental Sciences, Seoul National University, 08826 Seoul, South Korea.

[3]Univ. Grenoble-Alpes, CNRS, IRD, IGE, 38000 Grenoble, France.

[4]Division of atmospheric sciences, University of Helsinki, 00014 Helsinki, Finland.

[5]Institute of Atmospheric Science and Climate (ISAC)-CNR, 40129 Bologna, Italy.

* Corresponding author: meehye@korea.ac.kr

Supplementary Information Contents:

In total 19 pages including:

Text S1: Aerosol measurements (page 3).

Text S2: Aerosol chemical analysis (page 3).

Text S3: Retrieval and correction of aerosol optical properties (pages 3-4).

Text S4: Attribution of light absorption for BC, dust, and BrC and estimation of their MACs (pages 4-6).

Text S5: Seasonal categorization (page 6)

Figure S1-S3: 3 figures (pages 7-16)

Table S1: Seasonal characteristics of primary gases and water-soluble calcium levels (page 16)

References: References for the supplementary information (pages 17-19)

Text S1 Aerosol measurements.

The chemical compositions of PM_1, $PM_{2.5}$, and PM_{10} were measured during 2008-2010 at Gosan Climate Observatory (GCO, 33.17°N, 126.10°E, 70 m ASL; black star in Fig. S1) on Juju island. Aerosol samples were collected daily on pre-weighed 37-mm Teflon filters (Pall corp., USA) for mass and water-soluble ion analysis and on pre-heated 37-mm quartz-fiber filters (Pall corp., USA) for carbon analysis through PM_1, $PM_{2.5}$, and PM_{10} sharp-cut cyclones installed at the top of a 10-m high tower. Daily sampling (totaling 105 sets) was conducted usually once every six to eight days; however, additional samples were taken during particular events or samples were not taken during rainy season. For in-situ ambient aerosol measurements without size cut, aerosol absorption coefficients were recorded at seven wavelengths, 370, 450, 520, 590, 660, 880, and 950 nm, using an aethalometer (AE-31, Magee Scientific Corp., USA) every 5 minutes, and aerosol scattering coefficients were obtained at three wavelengths, 450 nm, 550 nm, and 700 nm, by an integrating nephelometer (model 3563, TSI Inc., USA) every 5 minutes. Gaseous pollutants, including O_3, NO_2, CO, and SO_2, and a meteorological suite were measured hourly by the National Institute of Environmental Research (NIER) and the Korea Meteorological Administration (KMA), respectively. These data were averaged hourly or daily for comparison with chemical composition data.

Text S2 Aerosol chemical analysis.

Eight species of water-soluble ions including SO_4^{2-}, NO_3^-, Cl^-, NH_4^+, K^+, Na^+, Ca^{2+}, and Mg^{2+} were determined by ion chromatography (Dionex 4500, Dionex, USA)[1,2]. The range of the detection limit (DL, 3σ) for each ion was 0.01–0.09 µg m^{-3}. Carbonaceous components including total carbon (TC), EC, and OC were analyzed at the Desert Research Institute (Reno, NV, USA) following the Interagency Monitoring of Protected Visual Environments thermal/optical reflectance protocol (i.e., IMP_TOR)[3]. The IMP_TOR method assumes that EC is a low-volatility carbon fraction that is not liberated in an oxygen-free environment until a temperature of >550°C is attained, allowing it to be separated from the more volatile OC that evolves at lower temperatures. Detailed information for the IMP_TOR method can be found in our companion papers[1,2]. The DLs for OC and EC in this study were 0.39 µg cm^{-2} and 0.01 µg cm^{-2}, respectively.

Text S3 Retrieval and correction of aerosol optical properties.

Aerosol light-absorption coefficients (σ_{ap}, Mm^{-1}) and scattering coefficients (σ_{sp}, Mm^{-1}) were corrected as shown below. The aethalometer measures light attenuation (ATN) and the attenuation coefficient (σ_{ATN}) can be calculated using the given sampling information[4]. This instrument needs to be corrected for the most significant biases of filter–particle interactions, such as scattering by fiber substrates of the filter (positive bias), scattering of aerosols embedded in the filter, and filter loading

by accumulation of light-absorbing particles (i.e., shadowing effect; negative bias)[5–7]. In this study, we basically adopted the way introduced by a previous study[7] to calculate σ_{ap} (Eq. 1) and for the aethalometer multi-scattering correction factor (hereafter denoted by C) we performed a field calibration by comparing with a Photoacoustic Spectrometer 3-wavelength (PASS-3; 405, 532, and 781 nm) (Eq. 2).

$\sigma_{ap} = \sigma_{ATN} / C \cdot R\,(\mathrm{ATN})$ [7], (1)

where C is the aethalometer multi-scattering correction factor and R(ATN) (≤1) explains the shadowing effect. Since the shadowing factor (R) is small for lightly loaded filters (ATN < 10), C can be determined as follows:

$C = \sigma_{ATN,\,10} / \sigma_{ap,\,PASS\text{-}3}$ [6], (2)

where $\sigma_{ATN,10}$ is σ_{ATN} of aethalometer when the ATN does not exceed 10%, and $\sigma_{ap,\,PASS\text{-}3}$ is σ_{ap} measured by PASS-3. Finally, the determined C values of 4.04 ± 1.68 and 4.89 ± 0.57 at 532 nm and 781 nm, respectively, were adjusted to seven wavelengths of the aethalometer by fitting power laws, 3.37, 3.80, 3.99, 4.25, 4.49, 5.18, and 5.39, at wavelengths from 370 nm to 950 nm. The σ_{sp} was corrected for truncation error[8].

Corrected σ_{ap} values were then used to calculate the absorption Å ngström exponent (AAE) of aerosols.

$\mathrm{AAE} = -\ln(\sigma_{ap}(\lambda_1)/\sigma_{ap}(\lambda_2))/\ln(\lambda_1/\lambda_2)$, (3)

Single scattering albedo (SSA; defined as $\sigma_{sp} / (\sigma_{ap}+\sigma_{sp})$) was calculated over the entire aethalometer wavelength range by fitting power law for σ_{sp}. The optical properties were averaged daily for comparison with the chemical compositions which were measured daily.

Text S4 Attribution of light absorption for BC, dust, and BrC and estimation of their MACs.

When deriving the mass absorption coefficient (MAC) for black carbon (BC) and brown carbon (BrC), we adopted the AAE of 1 for BC (AAE_{BC} of 1) to extrapolate BC absorption at 950 nm to shorter wavelengths as previously done by previous studies[9,10]. Although AAE_{BC} has recently been reported to be smaller than 1 in few calculations[11] and measurement studies[12], our measurement of AAE at 370 nm–950 nm (AAE) was always greater than 1, even under background conditions. The absorption of BC was normalized by PM_1 EC mass concentration, resulting in MAC of BC (MAC_{BC}) at all

wavelengths for each daily sample. We then estimated dust absorption, using a series of MAC of dust (MAC_{dust}; 0.087, 0.050, 0.037, 0.027, 0.013, 0.001 m^2 g^{-1} at 370 nm–880 nm), which was estimated for dust events at a polluted site near Beijing using the absorption predicted by Mie scattering theory and measured dust volume concentration[10]. Detailed information about the MAC_{dust} estimation is as follows. To estimate the MAC_{dust}, they first estimated the mass concentration and light absorption of dust from the supermicron portion of the size-distributed number concentrations from the aerodynamic particle sizer (APS) using Mie scattering theory. The refractive index for dust particles required in Mie calculation was determined using data during identified end-member dust events (see Table 1 in the original paper for the detail of identification for dust events) by varying imaginary component of the refractive index from 0i to 0.01i at each aethalometer wavelength, while keeping the real component constant at 1.53. It was assumed that absorption other than BC absorption was solely due to dust at those times. The least-square-fit method yielded an imaginary component of 0.0056i, 0.0033i, 0.0026i, 0.0019i, 0.001i, 0.0001i, and 0i at 370, 470, 520, 590, 660, 880, and 950 nm, respectively, by considering only when the square of the difference between the absorption other than BC absorption and Mie-predicted absorption was the smallest on average during the dust events. By assuming that all coarse particles had the same optical properties, this set of refractive indices was applied to all supermicron particle number concentrations to obtain a time-series of dust absorption for the whole measurement periods. The MAC_{dust} values were finally determined at each wavelength by normalizing the dust absorption by the mass concentration of dust aerosols, which was calculated from supermicron particle volume from the APS using a constant density of 2.6 g cm^{-3}.

This approach allowed us to apply the fixed MAC_{dust} for attributing dust absorption under the following assumptions: (i) the MAC_{dust} at near-UV and visible wavelengths is lower than that of OC by one or two orders of magnitude[10,13], (ii) the origins of the dust reaching GCO are the same as those of dust collected near Beijing, and (iii) the changes in the optical properties of dust due to transport and aging are negligible relative to the light-absorbing OC, which varies largely by an order of magnitude[14–16]. The dust mass concentration is estimated by supermicron (PM_{1-10}) water-soluble calcium (ws Ca^{2+}), using an Al/dust ratio in a Chinese loess-certified reference material (CRM) and a ws Ca^{2+}/Al (=0.24) ratio reported in a previous experiment conducted at GCO[17]. Using the fixed MAC_{dust} and the dust mass concentration, we estimated the dust absorption at each wavelength as dust absorption = dust mass concentration x MAC_{dust}. We then derived the BrC absorption by subtracting the absorption of BC and dust from the total absorption at all wavelengths, and the absorption of BrC was normalized by PM_1 OC mass concentration, resulting in MAC of BrC (MAC_{BrC}) at all wavelengths for each daily sample. The calculated BrC absorption at 370 nm was 3.1 ± 2.0 Mm^{-1} on average, corresponding to 20% of the total absorption. The mean MAC_{BrC} at 370 nm ($MAC_{BrC,370}$) was

1.2 m^2 g^{-1} at 370 nm, and it was 0.58 m^2 g^{-1} at 520 nm accounting for 10% of MAC_{BC}. The AAE of non-BC absorbing particles ($AAE_{non\text{-}BC}$; non-BC absorbing particles are sum of dust and BrC particles) was calculated at 370 nm–950 nm by subtracting the BC absorption from total absorption.

Text S5 Seasonal categorization.

Fig. S1 shows monthly NCEP/NCAR reanalysis wind vector (m s^{-1}) and geopotential height at 850 hPa together with endpoints of 72-h backward air mass trajectories over East Asia in 2008–2010. Changes in synoptic weather patterns are distinct for each season[18] and accordingly, regional-scale emission sources vary seasonally. High northwesterly winds prevail from October to March (cold season), resulting in the strongest continental outflow. The northwesterly wind weakens in April and May (spring). It should be noted that at GCO, March is often under a fast-moving synoptic weather pattern with northwesterly winds, which is more similar to the cold season weather pattern than it is to the spring pattern; therefore, we classified March as the cold season in this study. From June to September, the GCO site is under the influence of marine air masses with a prevailing southerly or easterly wind, which results in the lowest contribution of the continental emissions to GCO. Based on the seasonally distinctive synoptic weather patterns, our measurement data are divided into three seasonal groups: Cold (from October to March) and Warm (April and May [spring] and June to September [summer]). Accordingly, the levels of primary emissions such as CO, SO_2, and water-soluble calcium are summarized in Table S1 by season. Both primary gases and water-soluble calcium are at their maxima in the cold season and their minima in summer, which is consistent with the regional-scale weather patterns.

Supplementary Figures

a

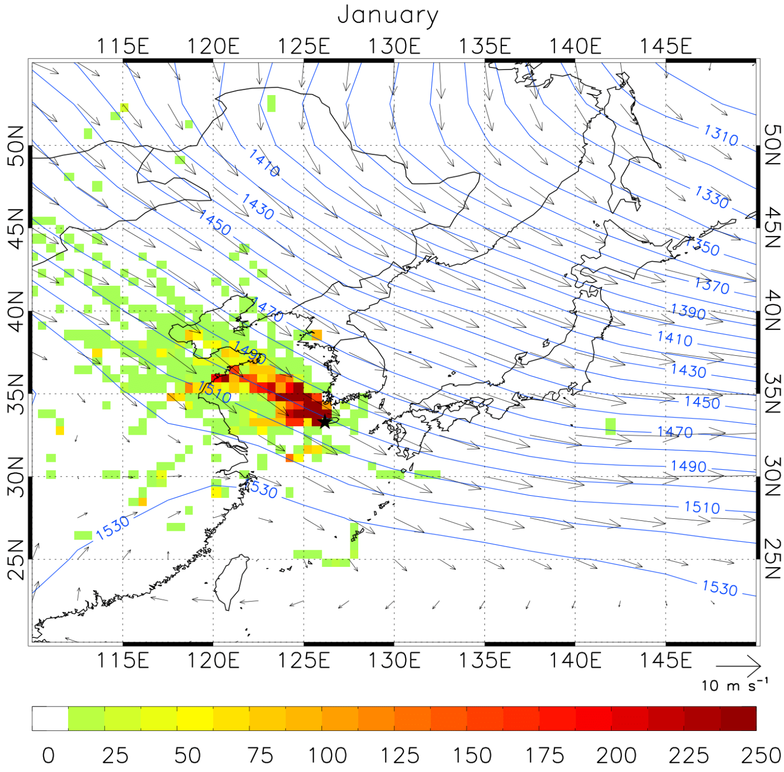

b

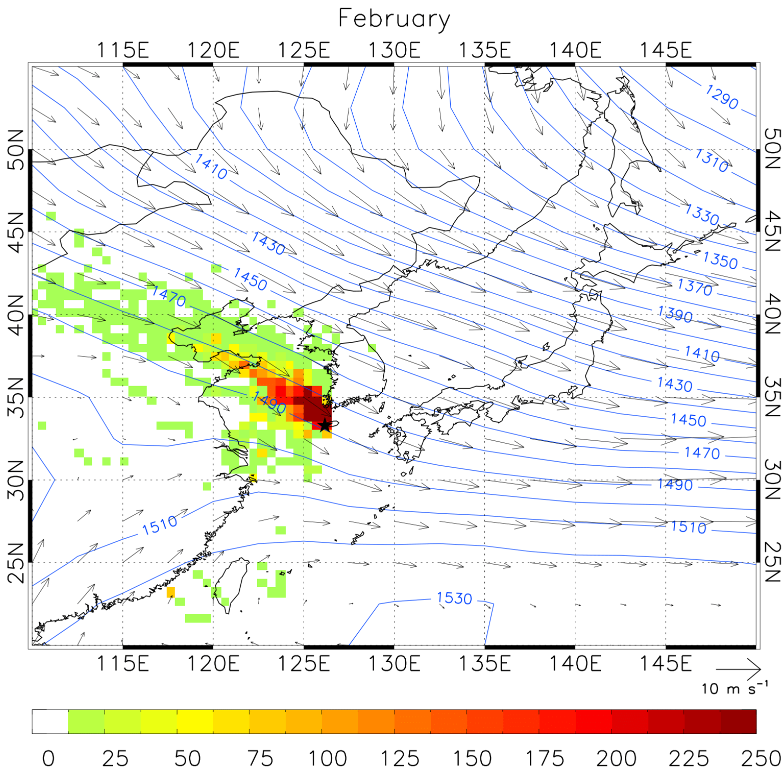
February
115E
120E
125E
130E
135E
140E
145E
50N
45N
40N
35N
30N
25N
10 m s-1
0
25
50
75
100
125
150
175
200
225
250

c

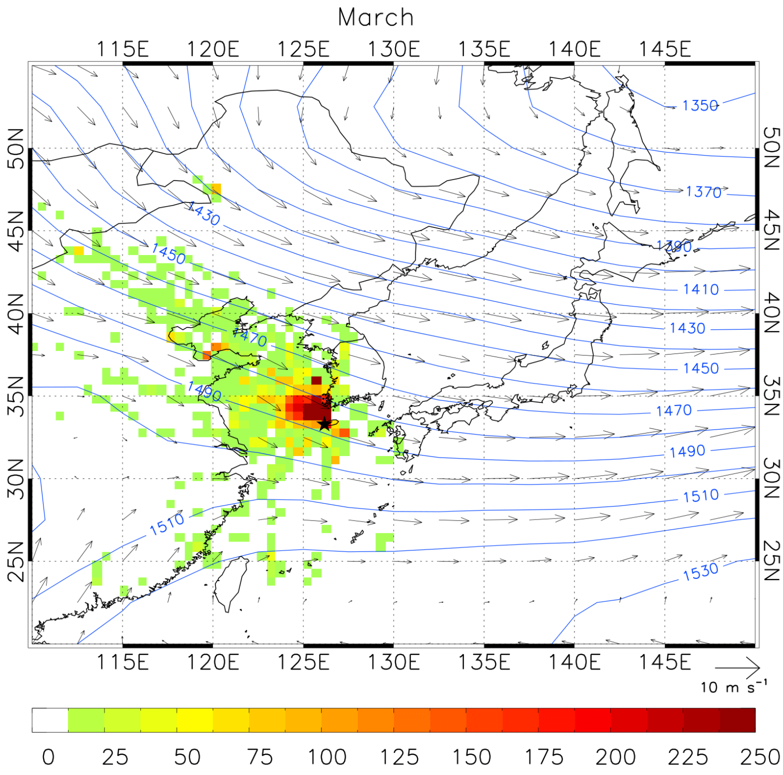
March
115E
120E
125E
130E
135E
140E
145E
50N
45N
40N
35N
30N
25N
10 m s-1
0
25
50
75
100
125
150
175
200
225
250

d

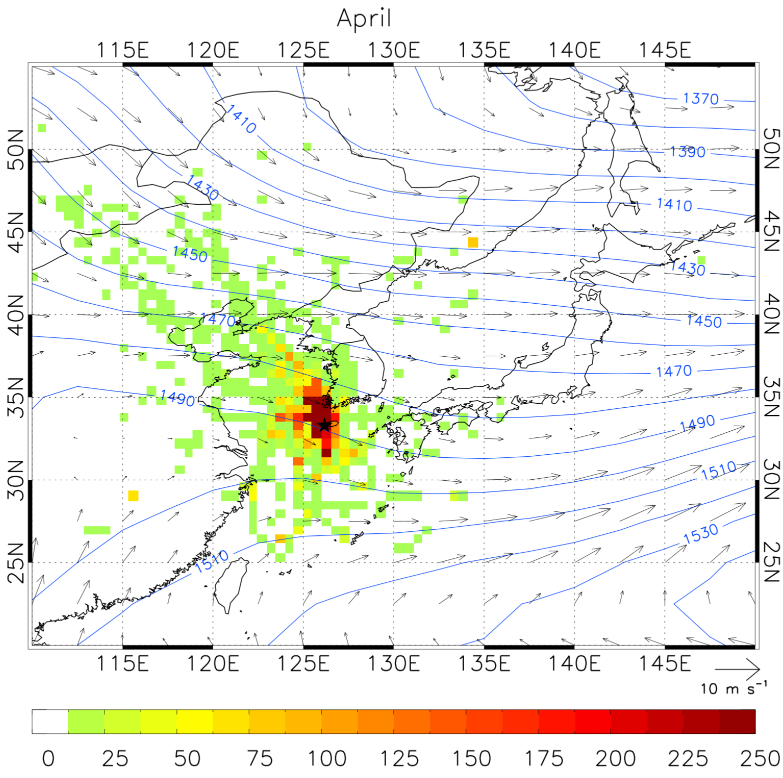
April
115E
120E
125E
130E
135E
140E
145E
50N
45N
40N
35N
30N
25N
1370
1390
1410
1430
1450
1470
1490
1510
1530
10 m s⁻¹
0
25
50
75
100
125
150
175
200
225
250

e

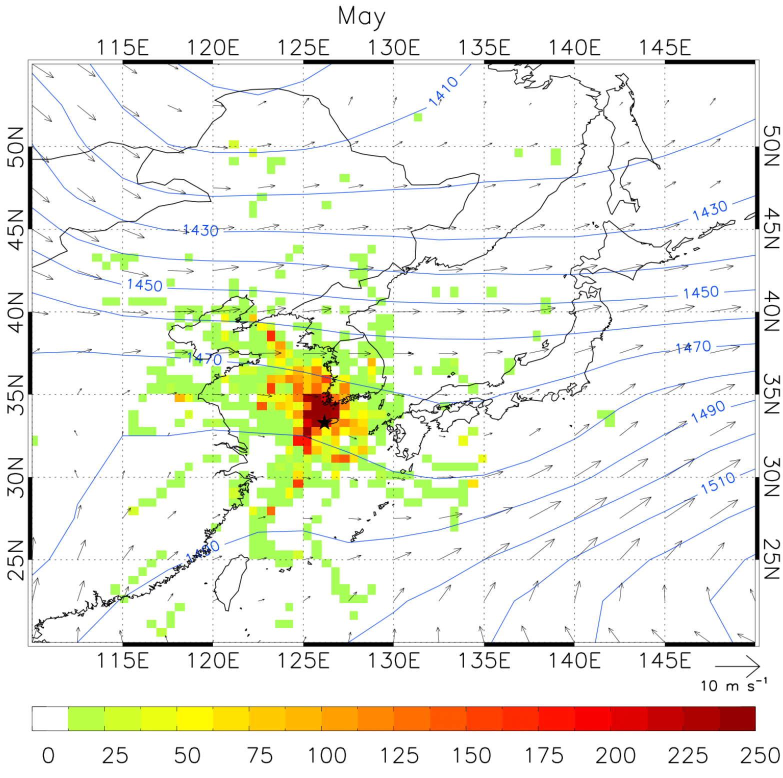
May
115E
120E
125E
130E
135E
140E
145E
50N
45N
40N
35N
30N
25N
1410
1430
1450
1470
1490
1510
10 m s⁻¹
0
25
50
75
100
125
150
175
200
225
250

f

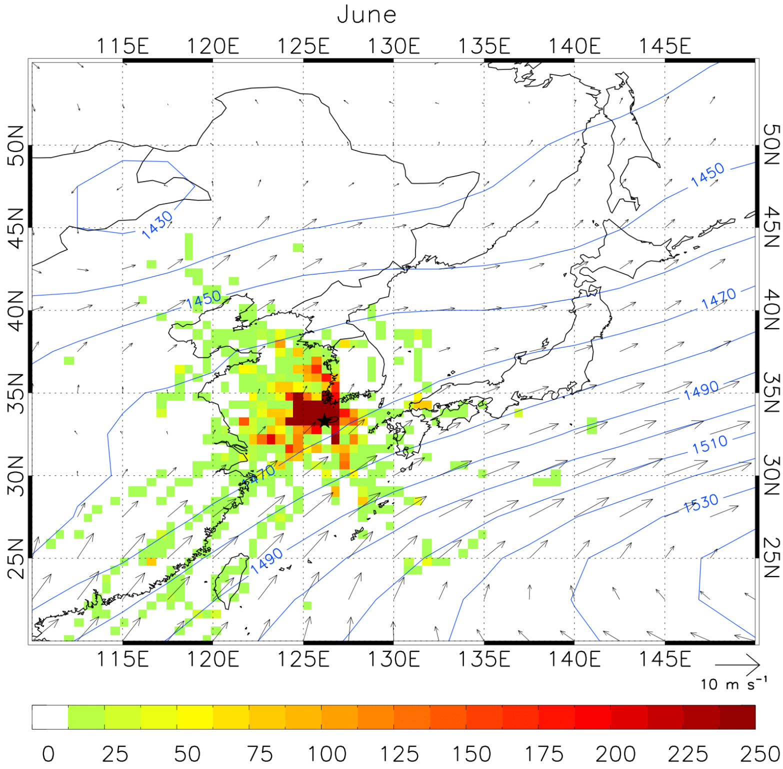
June
115E 120E 125E 130E 135E 140E 145E
50N
45N
40N
35N
30N
25N
1430
1450
1470
1490
1510
1530
10 m s-1
0 25 50 75 100 125 150 175 200 225 250

g

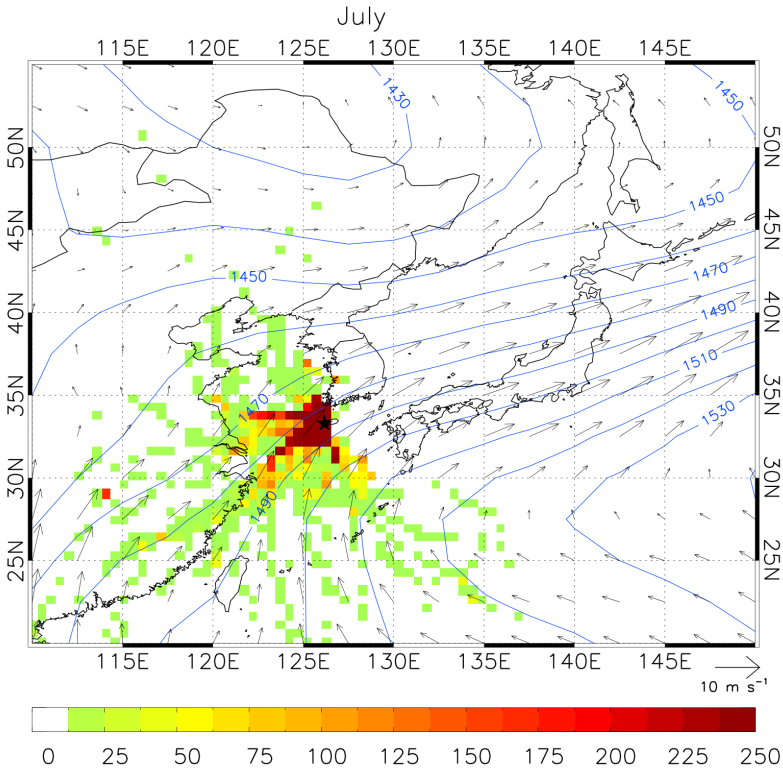
July
115E 120E 125E 130E 135E 140E 145E
50N
45N
40N
35N
30N
25N
1430
1450
1470
1490
1510
1530
10 m s-1
0 25 50 75 100 125 150 175 200 225 250

h

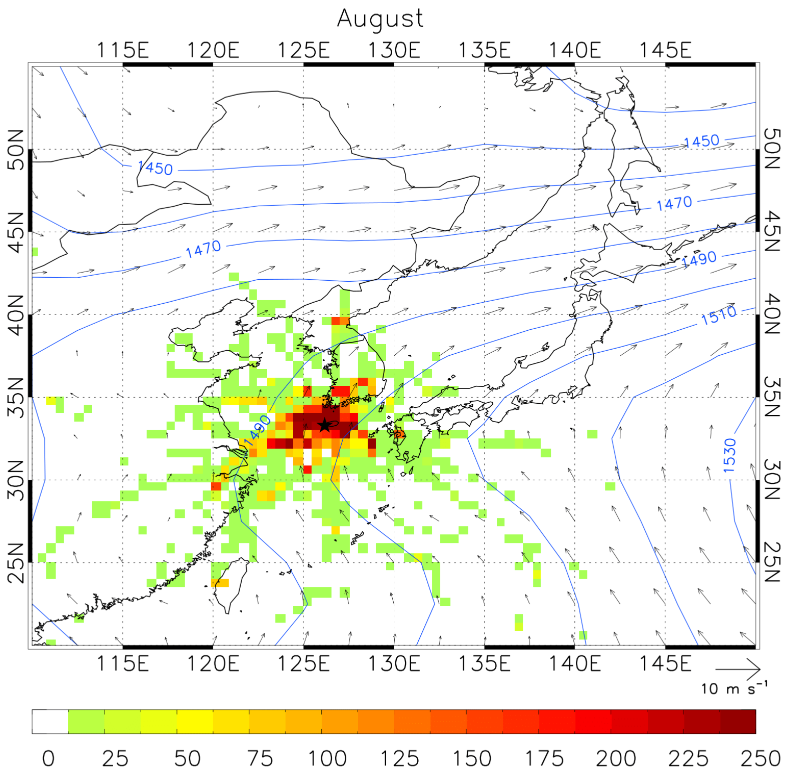
August
115E
120E
125E
130E
135E
140E
145E
50N
45N
40N
35N
30N
25N
1450
1470
1490
1510
1530
10 m s⁻¹
0
25
50
75
100
125
150
175
200
225
250

i

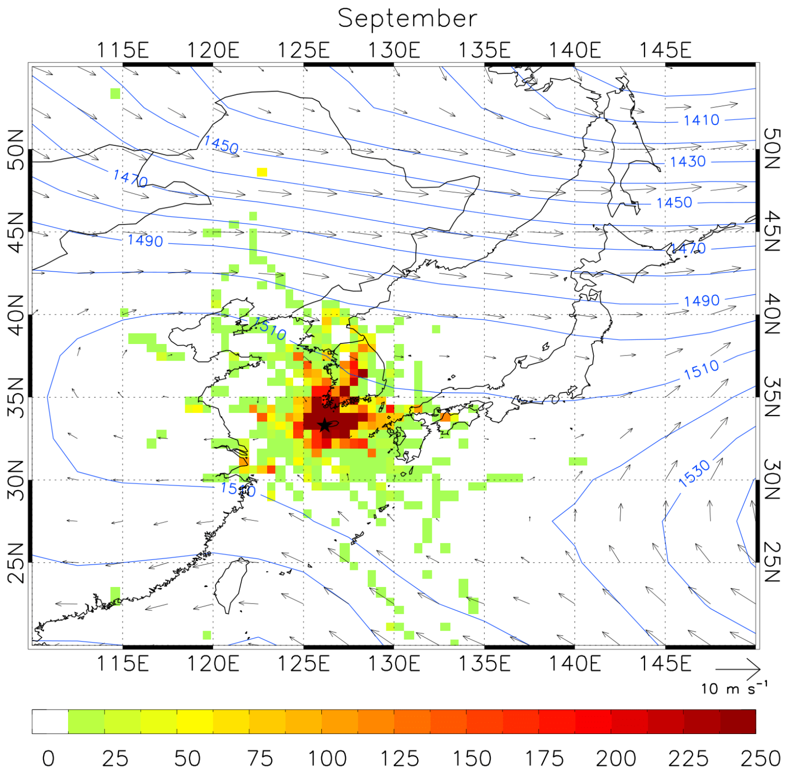
September
115E
120E
125E
130E
135E
140E
145E
50N
45N
40N
35N
30N
25N
1410
1430
1450
1470
1490
1510
1530
10 m s⁻¹
0
25
50
75
100
125
150
175
200
225
250

j

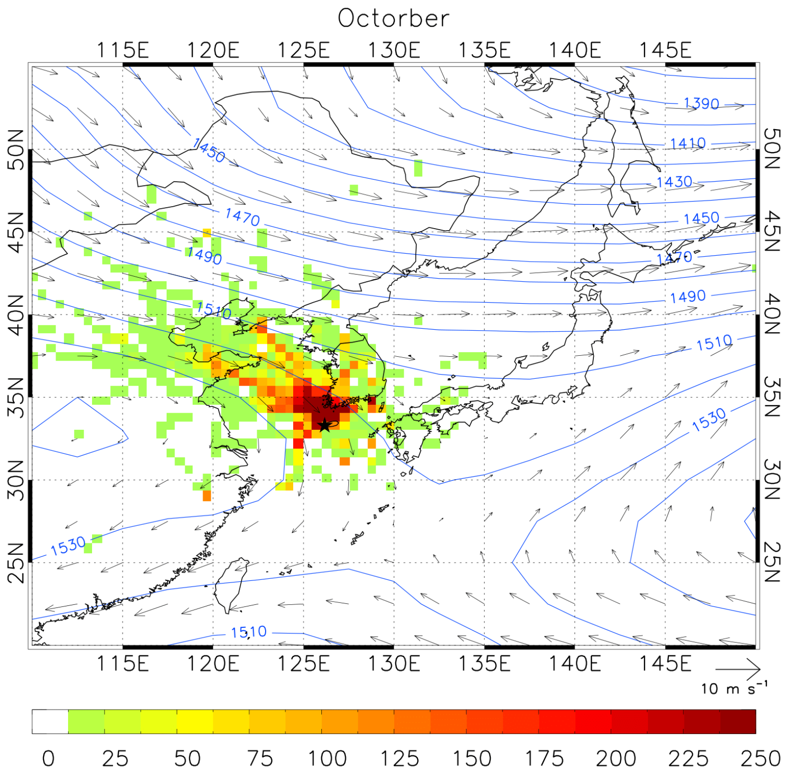
Octorber
115E 120E 125E 130E 135E 140E 145E
50N 45N 40N 35N 30N 25N
1390
1410
1430
1450
1470
1490
1510
1530
10 m s^{-1}
0 25 50 75 100 125 150 175 200 225 250

k

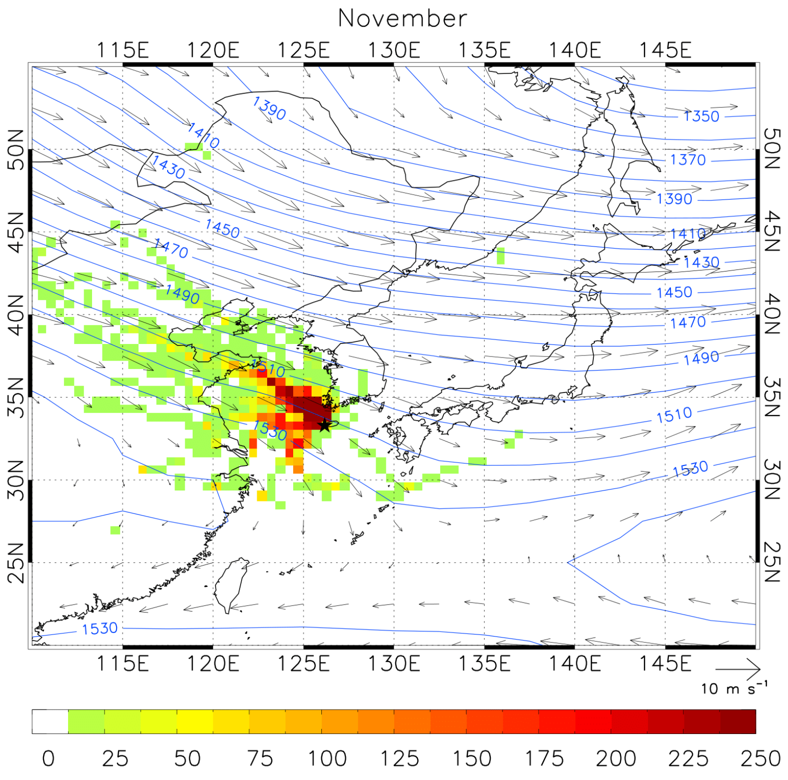
November
115E 120E 125E 130E 135E 140E 145E
50N 45N 40N 35N 30N 25N
1350
1370
1390
1410
1430
1450
1470
1490
1510
1530
10 m s^{-1}
0 25 50 75 100 125 150 175 200 225 250

l

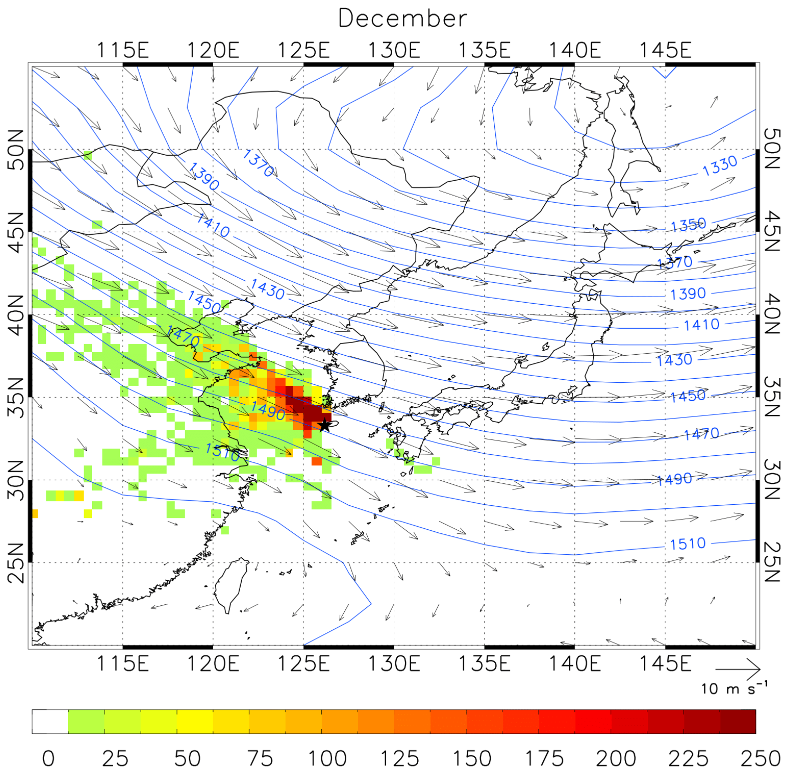

Figure S1. Monthly reanalysis wind vector with geopotential height at 850 hPa and endpoints of 72-h backward air mass trajectories during 2008–2010. From (a) January to (l) December. The location of GCO is marked in black star. The wind vector data was provided from NCEP-DOE Reanalysis 2[19]. The air mass trajectories were calculated using the HYSPLIT model[20,21] with the global data assimilation system (GDAS) meteorological input at 24hrs interval. HYSPLIT: hybrid single particle Lagrangian Integrated trajectory model. Maps were created using IDL (Version 7.1 (c) 2009, ITT Exelis Visual Information Solutions, Inc.).

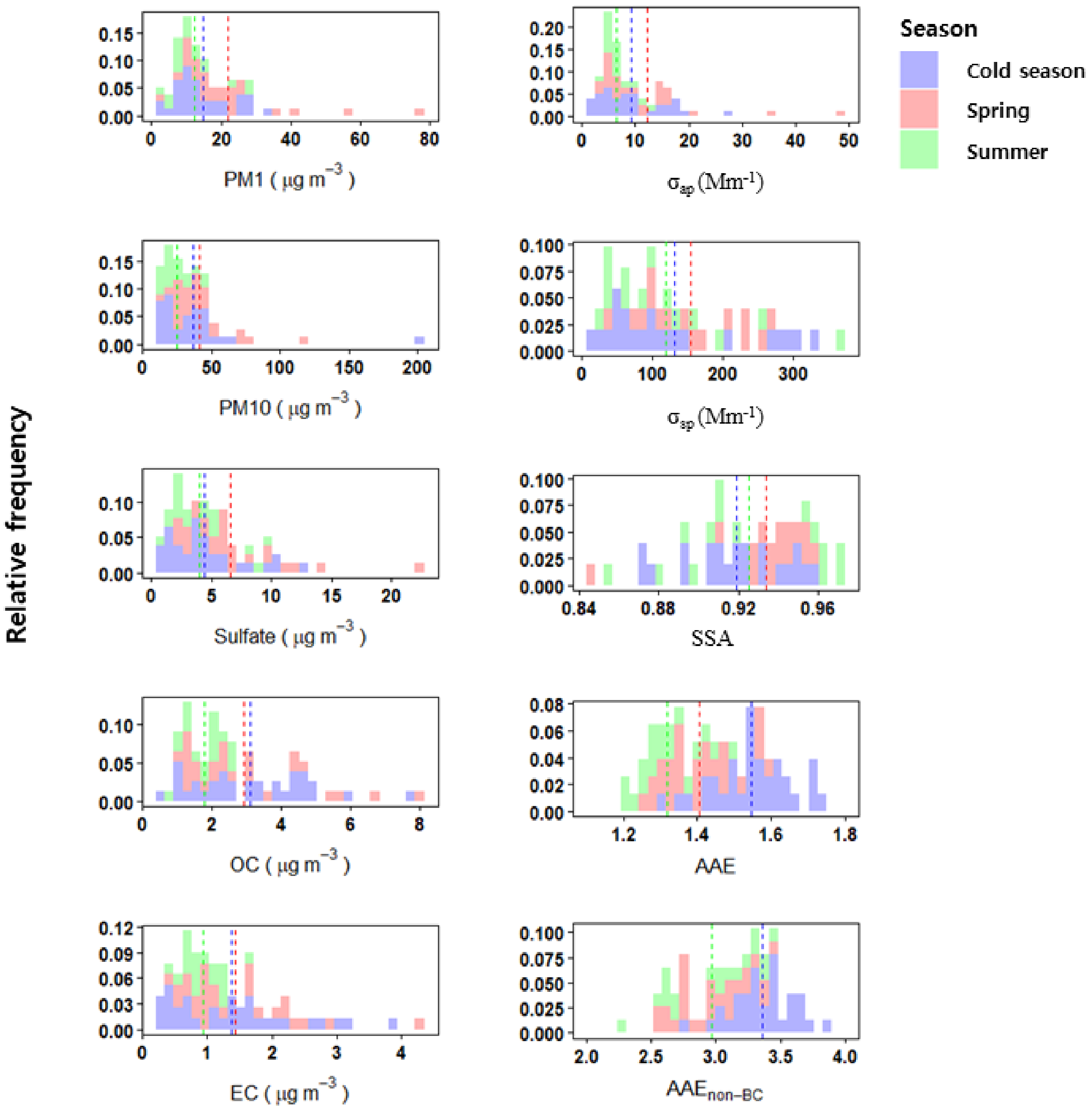

Figure S2. Seasonal frequency distributions of chemical properties (left panel; PM_1, PM_{10}, and sulfate, OC, and EC in PM_1) and optical properties (right panel; σ_{ap}, σ_{sp}, SSA, AAE, and $AAE_{non\text{-}BC}$). σ_{ap}, σ_{sp}, and SSA are at 520 nm, and AAE and $AAE_{non\text{-}BC}$ are from 7 wavelengths between 370 nm–950 nm. Vertical dot lines indicate seasonal means.

a

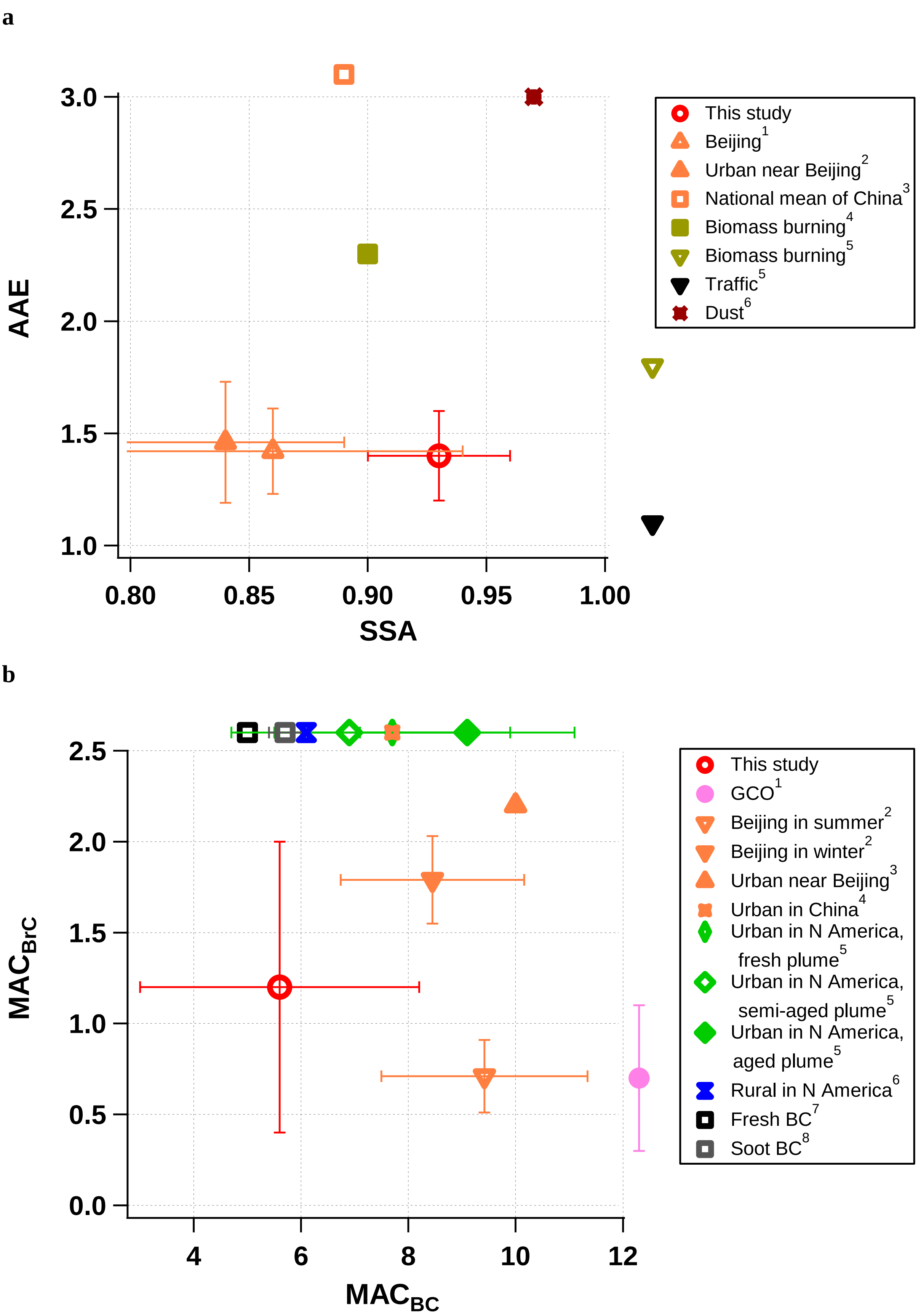

Figure S3. Comparison of aerosol optical properties of this study (red open circles) with

those of other studies (other marks). a, AAEs versus SSAs. Reported AAE values were measured mostly from near-UV to near-IR spectrums and SSA values in the visible spectrum[22,10,23,24,25,26 (in order)]; **b**, Mass absorption cross-section of brown carbon (MAC_{BrC}) versus mass absorption cross-section of BC (MAC_{BC}). Reported MAC_{BC} and MAC_{BrC} were derived in the visible spectrum and the near-UV spectrum, respectively[16 27,10,28,29,30,31,32 (in order)]. MAC_{BC} and MAC_{BrC} in this study are at 520 nm and 370 nm, respectively. In **a** and **b**, symbols plotted on the right and top of the graph denote no-paired measurements of SSA or AAE and MAC_{BC} or MAC_{BrC}, respectively.

Table S1. Seasonal characteristics of primary gases and water-soluble calcium levels.

	**CO (ppbv)**		**SO_2 (ppbv)**		**PM_{10} Ca^{2+} (μg m^{-3})**	
**Season (month)**	**Mean**	**Sd**	**Mean**	**Sd**	**Mean**	**Sd**
**Cold season (Oct.–Mar.)**	613.8	233.6	4.1	2.9	0.41	0.50
**Spring (Apr.-May)**	570.3	246.3	3.4	2.1	0.42	0.27
**Summer (Jun.–Sep.)**	509.2	163.3	1.6	0.8	0.27	0.44

Acknowledgements

The authors gratefully acknowledge the NOAA Air Resources Laboratory (ARL) for the provision of the HYSPLIT transport and dispersion model and/or READY website (http://www.ready.noaa.gov) used in this publication.

Supplementary References

1. Lim, S. *et al.* Ionic and carbonaceous compositions of PM10, PM2.5 and PM1.0 at Gosan ABC Superstation and their ratios as source signature. *Atmos. Chem. Phys.* **12,** 2007–2024 (2012).
2. Lim, S. *et al.* Absorption and scattering properties of organic carbon versus sulfate dominant aerosols at Gosan climate observatory in Northeast Asia. *Atmos. Chem. Phys.* **14,** 7781–7793 (2014).
3. Chow, J. C. *et al.* The dri thermal/optical reflectance carbon analysis system: description, evaluation and applications in U.S. Air quality studies. *Atmos. Environ. Part A. Gen. Top.* **27,** 1185–1201 (1993).
4. Hansen, A. D. A. *Aethalometer-User Manual*. (2005).
5. Cllaud Coen, M. *et al.* Minimizing light absorption measurement artifacts of the Aethalometer: evaluation of five correction algorithms. *Atmos. Meas. Tech.* **3,** 457–474 (2010).
6. Schmid, O. *et al.* Spectral light absorption by ambient aerosols influenced by biomass burning in the Amazon Basin. I: Comparison and field calibration of absorption measurement techniques. *Atmos. Chem. Phys.* **6,** 3443–3462 (2006).
7. Weingartner, E. *et al.* Absorption of light by soot particles: determination of the absorption coefficient by means of aethalometers. *J. Aerosol Sci.* **34,** 1445–1463 (2003).
8. Anderson, T. L. & Ogren, J. A. Determining Aerosol Radiative Properties Using the TSI 3563 Integrating Nephelometer. *Aerosol Sci. Technol.* **29,** 57–69 (1998).
9. Clarke, A. *et al.* Biomass burning and pollution aerosol over North America: Organic components and their influence on spectral optical properties and humidification response. *J. Geophys. Res.* **112,** D12S18 (2007).
10. Yang, M., Howell, S. G., Zhuang, J. & Huebert, B. J. Attribution of aerosol light absorption to black carbon, brown carbon, and dust in China – interpretations of

atmospheric measurements during EAST-AIRE. *Atmos. Chem. Phys.* **9,** 2035–2050 (2009).

11. Lack, D. A. & Langridge, J. M. On the attribution of black and brown carbon light absorption using the Å ngström exponent. *Atmos. Chem. Phys.* **13,** 10535–10543 (2013).
12. Yuan, J.-F. *et al.* Light absorption of brown carbon aerosol in the PRD region of China. *Atmos. Chem. Phys.* **16,** 1433–1443 (2016).
13. Favez, O., Alfaro, S. C., Sciare, J., Cachier, H. & Abdelwahab, M. M. Ambient measurements of light-absorption by agricultural waste burning organic aerosols. *J. Aerosol Sci.* **40,** 613–620 (2009).
14. Alexander, D. T. L., Crozier, P. A. & Anderson, J. R. Brown carbon spheres in East Asian outflow and their optical properties. *Science* **321,** 833–6 (2008).
15. Kirchstetter, T. W., Novakov, T. & Hobbs, P. V. Evidence that the spectral dependence of light absorption by aerosols is affected by organic carbon. *J. Geophys. Res. Atmos.* **109,** D21208 (2004).
16. Kirillova, E. N., Andersson, A., Han, J., Lee, M. & Gustafsson, Ö . Sources and light absorption of water-soluble organic carbon aerosols in the outflow from northern China. *Atmos. Chem. Phys.* **14,** 1413–1422 (2014).
17. Arimoto, R. Chemical composition of atmospheric aerosols from Zhenbeitai, China, and Gosan, South Korea, during ACE-Asia. *J. Geophys. Res.* **109,** D19S04 (2004).
18. Kim, S.-W., Yoon, S.-C., Kim, J. & Kim, S.-Y. Seasonal and monthly variations of columnar aerosol optical properties over east Asia determined from multi-year MODIS, LIDAR, and AERONET Sun/sky radiometer measurements. *Atmos. Environ.* **41,** 1634–1651 (2007).
19. Kanamitsu, M. *et al.* NCEP-DOE AMIP-II Reanalysis (R-2). *Bull. Am. Meteorol. Soc.* 1631–1643 (2002).
    Websites: https://www.esrl.noaa.gov/psd/data/gridded/data.ncep.reanalysis2.html, http://www.cpc.ncep.noaa.gov/products/wesley/reanalysis2/kana/reanl2-1.htm.
20. Draxler, R. R. & Hess, G. . An overviewof theHYSPLIT_4modelling system for trajectories, dispersion and deposition. *Aust. Meteorol. Mag.* **47,** 295–308 (1998).
    Website: https://www.arl.noaa.gov/ready/hysplit4.html.
21. Stein, A. F. *et al.* NOAA’s HYSPLIT Atmospheric Transport and Dispersion

Modeling System. *Bull. Am. Meteorol. Soc.* **96,** 2059–2077 (2015).

22. Garland, R. M. *et al.* Aerosol optical properties observed during Campaign of Air Quality Research in Beijing 2006 (CAREBeijing-2006): Characteristic differences between the inflow and outflow of Beijing city air. *J. Geophys. Res.* **114,** D00G04 (2009).
23. Lee, K. H. *et al.* Aerosol single scattering albedo estimated across China from a combination of ground and satellite measurements. *J. Geophys. Res.* **112,** D22S15 (2007).
24. Lack, D. a *et al.* Brown carbon and internal mixing in biomass burning particles. *Proc. Natl. Acad. Sci. U. S. A.* **109,** 14802–7 (2012).
25. Sandradewi, J. *et al.* A study of wood burning and traffic aerosols in an Alpine valley using a multi-wavelength Aethalometer. *Atmos. Environ.* **42,** 101–112 (2008).
26. Alfaro, S. C. Iron oxides and light absorption by pure desert dust: An experimental study. *J. Geophys. Res.* **109,** D08208 (2004).
27. Cheng, Y. *et al.* Mass absorption efficiency of elemental carbon and water-soluble organic carbon in Beijing, China. *Atmos. Chem. Phys.* **11,** 11497–11510 (2011).
28. Andreae, M. O. *et al.* Optical properties and chemical composition of the atmospheric aerosol in urban Guangzhou, China. *Atmos. Environ.* **42,** 6335–6350 (2008).
29. Knox, A. *et al.* Mass Absorption Cross-Section of Ambient Black Carbon Aerosol in Relation to Chemical Age. *Aerosol Sci. Technol.* **43,** 522–532 (2009).
30. Quincey, P., Butterfield, D., Green, D., Coyle, M. & Cape, J. N. An evaluation of measurement methods for organic, elemental and black carbon in ambient air monitoring sites. *Atmos. Environ.* **43,** 5085–5091 (2009).
31. Bond, T. C. *et al.* Bounding the role of black carbon in the climate system: A scientific assessment. *J. Geophys. Res. Atmos.* **118,** 5380–5552 (2013).
32. Schnaiter, M. *et al.* UV-VIS-NIR spectral optical properties of soot and soot-containing aerosols. *J. Aerosol Sci.* **34,** 1421–1444 (2003).
